# A Pilot Study on the Potential of RNA-Associated to Urinary Vesicles as a Suitable Non-Invasive Source for Diagnostic Purposes in Bladder Cancer

**DOI:** 10.3390/cancers6010179

**Published:** 2014-01-22

**Authors:** Amparo Perez, Ana Loizaga, Raquel Arceo, Isabel Lacasa, Ainara Rabade, Kerman Zorroza, David Mosen-Ansorena, Esperanza Gonzalez, Ana M. Aransay, Juan M. Falcon-Perez, Miguel Unda-Urzaiz, Felix Royo

**Affiliations:** 1 Urology Service, Basurto University Hospital, Bilbao 48013, Bizkaia, Spain; E-Mails: amparo.perezfernandez@osakidetza.net (A.P.); ana.loizagairiarte@osakidetza.net (A.L.); raquel.arceosantiago@osakidetza.net (R.A.); i_lacasa@hotmail.com (I.L.); ainarabade@hotmail.com (A.R.); jesusmiguel.undaurzaiz@osakidetza.net (M.U.-U.); 2 Basque Foundation for Health Innovation and Research (BIOEF), DNA Laboratory, Basurto Hospital, Bilbao 48013, Bizkaia, Spain; E-Mail: kerman.zorrozafernandez@osakidetza.net; 3 Genome Analysis Platform, CIC bioGUNE, CIBERehd, Bizkaia Technology Park, Derio 48160, Bizkaia, Spain; E-Mails: dmosen.ng@cicbiogune.es (D.M.-A); amaransay@cicbiogune.es (A.M.A.); 4 Metabolomics Unit, CIC bioGUNE, CIBERehd, Bizkaia Technology Park, Derio 48160, Bizkaia, Spain; E-Mails: egonzalez@cicbiogune.es (E.G.); jfalcon@cicbiogune.es (J.M.F.-P.); 5 IKERBASQUE, Basque Foundation for Science, Bilbao 48011, Bizkaia, Spain; E-Mail: jfalcon@ciberehd.es

**Keywords:** extracellular vesicles, bladder cancer, gene expression analysis, *LASS2*, *GALNT1*, *ARHGEF39*, *FOXO3*

## Abstract

Bladder cancer is one of the most common cancers and, together with prostate carcinoma, accounts for the majority of the malignancies of the genitourinary tract. Since prognosis ameliorates with early detection, it will be beneficial to have a repertoire of diagnostic markers that could complement the current diagnosis protocols. Recently, cell-secreted extracellular vesicles have received great interest as a source of low invasive disease biomarkers because they are found in many body fluids, including urine. The current work describes a pilot study to generate an array-based catalogue of mRNA associated to urinary vesicles, and also a comparison with samples obtained from bladder cancer patients. After an analysis of presence/absence of transcripts in bladder cancer EVs, a list of genes was selected for further validation using PCR technique. We found four genes differentially expressed in cancer samples. *LASS2* and *GALNT1* were present in cancer patients, while *ARHGEF39* and *FOXO3* were found only in non-cancer urinary vesicles. Previous studies have pointed to the involvement of those genes in tumour progression and metastasis.

## 1. Introduction

Cancer of the urinary bladder is among the five most common malignancies in the Western world [1,2] and the frequency of transitional cell carcinoma (TCC), which accounts for 90% of bladder cancers, is second only to prostate cancer as a malignancy of the genitourinary tract [3]. Among men, it is the fourth most common tumour, and it is the ninth leading cause of death from cancer. The ratio of men to women that develop bladder cancer is approximately 3:1 [3]. When first diagnosed, more than 80% of bladder tumours are non-muscle invasive papillary tumours that have an excellent prognosis. If left untreated, however, these initially non-muscle invasive lesions can progress to being muscle-invasive [4]. The remaining 20% exhibit muscle invasion at the time of diagnosis and have a much less favourable prognosis, including a 5% of patients with metastatic tumors [5]. Despite radical therapy, the mortality from invasive urothelial cell carcinoma is about 50%, reflecting the typical late stage at diagnosis and a poor response to chemotherapy [6]. It has been suggested that malignant tumors arise from a hyperplasic benign change and evolve toward a papillary neoplasm through angiogenetic responses [5]. These facts highlight the importance of an early diagnosis for the survival of cancer bladder patients.

The current standard of care for the detection of bladder cancer is based on flexible cystoscopy, usually combined with urine cytology. Cystoscopy is an invasive technique with a high sensitivity (91%) [7], although it can overlook flat malignancies such as carcinoma *in situ*. Cytology is a non-invasive technique with high specificity (90%–96%) [8], but it lacks sensitivity (11% to 76%) [9]. All these limitations of current diagnostic methods have prompted a search for additional markers. The high frequency of cell exfoliation into urine has promoted the study of gene expression in urine sediments. In fact, different tests based on genetic signatures in urine have been applied in cohort studies and are a realistic alternative for pre-screening [10,11].

The study of extracellular vesicles (EVs) has emerged in the last years as an alternative to identify non invasive biomarkers. Urine contains EVs [12] and, importantly, elevated levels of EVs have been found in urine samples from prostate cancer patients [13]. Moreover, EVs in bladder cancer patients have been characterized by their proteomic profile [14,15,16]. However, in spite of the fact that urinary EVs contain mRNA [17], there are no publications about the value of the transcriptome carried by urinary EVs for bladder cancer diagnostic and prognosis. Since certain RNA transcripts are enriched several fold in EVs compared with the donor cells [18], it is possible to speculate that EVs transcriptome may reveal tumour specific transcripts which would not be found in other urine fractions. This is supported by the fact that in previous studies, nucleic acid cargo of urinary EVs has been proven useful for diagnostic of myocardial infarction [19], prostate cancer [20] and liver damage [21]. Following this hypothesis, the present work is a comparative study of the transcriptomes carried within urinary vesicles obtained from bladder cancer and non-cancer patients. Using microarray technology, we assembled the first catalogue of mRNAs associated with urinary vesicles and identified candidate genes that could be useful as biomarkers for diagnosis and prognosis of bladder cancer. 

## 2. Experimental

### 2.1. Human Samples

The samples were obtained from patients with different urinary tract conditions, suffering different symptomatology, such as haematuria, dysuria or frequent urination, and were cited for exploratory cystoscopy. The study follows all the ethical regulations and was approved by the Basque Ethical Committee for Clinical Research (identification number CEIC 09-27). Previous to sample collection, patients were informed about the study and requested to sign the informed consent. For each sample, all the urine was collected by spontaneous micturition previous to cystoscopy, centrifuged at 2,000 × *g* for 10 min, filtered through a 0.22 μm pore membrane, and immediately frozen at −80 °C. The samples were classified as “cancer” when both cystoscopy and subsequent histological analysis were positive. Meanwhile, the control group included samples with negative results for both cystoscopy and cytology analyses.

### 2.2. Isolation of EVs

To purify EVs from urine, the stored samples were thawed, centrifuged at 2,000 × *g* for 10 min, and then ultracentrifuged at 100,000 × g for 75 min. The pellet was re-suspended in 150 uL of PBS and kept at −80 °C until processed. 

### 2.3. EVs Characterization

Size distribution within EVs preparations was analyzed by measuring the rate of Brownian motion using a NanoSight LM10 system (NanoSight, Amesbury, UK), which is equipped with a fast video capture and Nano-particle Tracking Analysis (NTA) software. NTA post-acquisition settings were kept constant for all samples, and each video was analyzed to give the mean, mode, and median vesicle size, as well as an estimate of the concentration [22]. For cryo-electron microscopy, extracellular vesicle preparations were directly adsorbed onto glow-discharged holey carbon grids (QUANTIFOIL Micro Tools GmbH, Jena, Germany). Grids were blotted at 95% humidity and rapidly plunged into liquid ethane with the aid of VITROBOT (Maastricht Instruments BV, Maastricht, The Netherlands). Vitrified samples were imaged at liquid nitrogen temperature using a JEM-2200FS/CR transmission cryo-electron microscope (JEOL, Tokio Japan) equipped with a field emission gun and operated at an acceleration voltage of 200 kV.

### 2.4. RNA Extraction

Total RNA isolation was achieved by RNeasy columns (Qiagen, Inc., Valencia, CA, USA). Their integrity, size and quantification were evaluated in RNA Pico Chips with a Bioanalyzer (Agilent Technologies, Santa Clara, CA, USA). 

### 2.5. Microarray Gene Expression Data

Whole genome expression characterization was conducted using Human HT12 v4 BeadChips (Illumina Inc., San Diego, CA, USA). cRNA synthesis was obtained with TargetAmp™ Nano-g™ Biotin-aRNA Labeling Kit for the Illumina^®^ System, Epicentre (Cat. Num. TAN07924) and subsequent amplification, labeling and hybridization were performed according to Whole-Genome Gene Expression Direct Hybridization Illumina Inc.’s protocol. Raw data were extracted with GenomeStudio analysis software (Illumina Inc.) in the form of GenomeStudio’s Final Report (sample probe profile).

In the R statistical computing environment, using the lumi Bioconductor package [23], raw data were background corrected, log2 transformed and quantile normalized.

For the calling of present transcripts, GenomeStudio provides detection *p*-values per probe and sample. We regarded a transcript as significantly present in a sample if its corresponding probe’s *p*-value was lower than 0.01. For the assessment of differentially detected transcripts, a linear model was fitted to the data and empirical Bayes moderated t-statistics were calculated using the limma package from Bioconductor [24].

### 2.6. Reverse Transcriptase and PCR

cDNA was synthesized from 0.1–1 ng of RNA using Superscript III (Invitrogen) following the manufacturer’s recommendations. Primers were designed using Primer3 [25], and the sequences are listed in Supplementary Table S1. To amplify targeted fragments and estimate relative quantification of genes, PCR was performed in duplicates using Quanta SYBR Green PCR Master Mix (Perfecta, Gaithersburg, MD, USA) in an iCycler thermocycler (BioRad Laboratories Inc., Hercules, CA, USA). To confirm the identity of amplicons, PCR products were run on agarose gels to confirm that their sizes matched with the designed reactions. 

## 3. Results

A total of five urine samples from bladder cancer patients (all males; age range: 42–86 years, mean: 64.40 and SD: 16.80) and six samples from non-cancer patients (all males; age range: 45–68 years, mean: 58.00 and SD: 9.59) were characterized by whole transcriptome arrays. An additional three cancer (1 male and 2 females; age range 64–86 years, mean: 71.33 and SD = 12.70) and five control (4 males and 1 female; age range 59–74 years, mean 68.20 and SD = 6.46) samples were considered for the validation step. 

**Figure 1:**
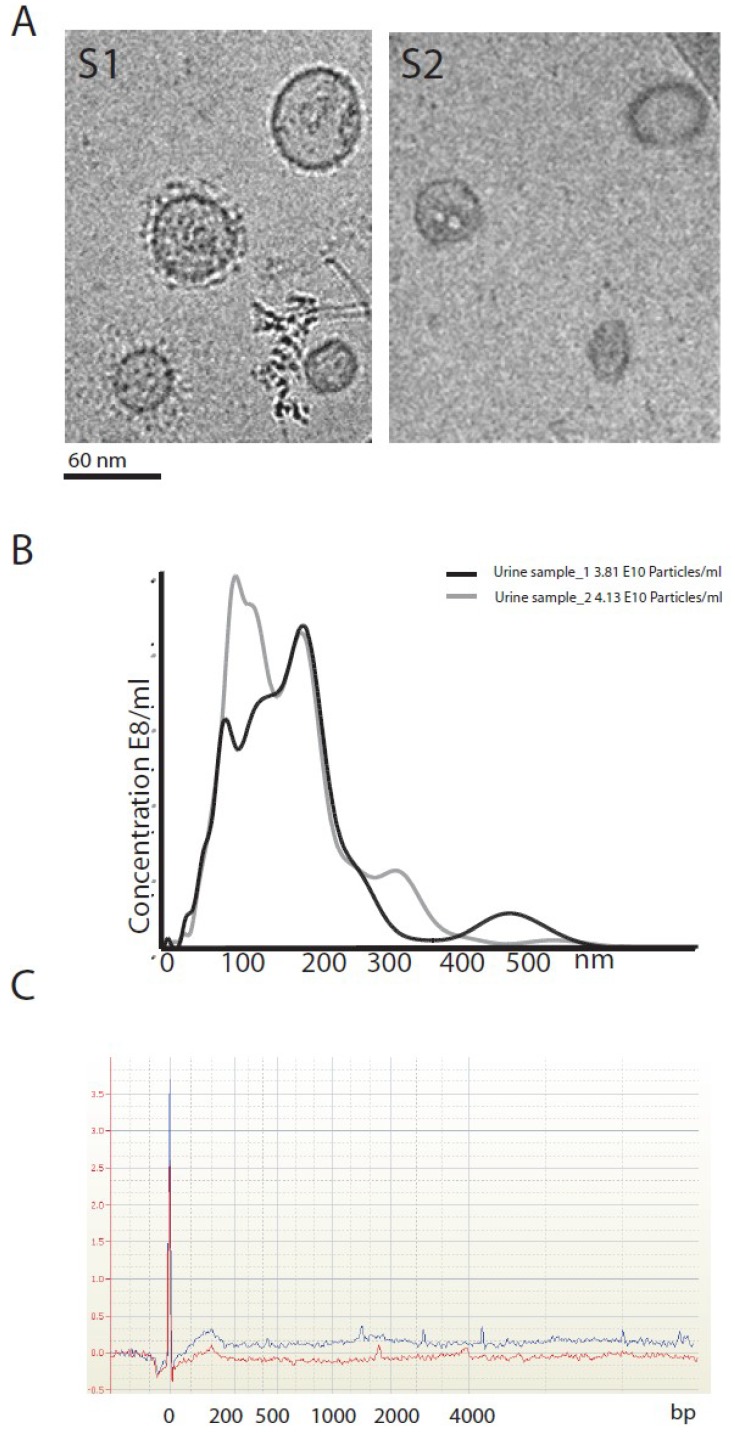
Characterization of EVs isolated from voided urine. Two different samples observed by electron microscopy image (**A**), size profile determined by NTA (**B**) and the RNA profile after RNA extraction from EVs (**C**).

We isolated urinary vesicles from urine samples and characterized them by electron microscopy and particle tracking analysis applying NTA techniques. Purified EVs observed under electron microscopy offered a heterogeneous morphology (Figure 1A). Accordingly, the size profiles obtained with NTA analysis differed slightly between samples (see two representative examples in Figure 1B). The RNA content isolated from the vesicles ranged from 50 pg to 30 ng, and the RNA size profiles indicated that fragments below 200 bp were enriched in EVs (Figure 1C).

Whole genome expression analysis detected 4,102 transcripts across the studied samples, which is about one tenth of the probes tested by the Human HT12 v4 BeadChips (Illumina Inc.). The data discussed in this publication have been deposited in NCBI’s Gene Expression Omnibus and are accessible through GEO Series accession number GSE51843) We observed a high variability among samples, with a very low correlation among them (Supplementary Figure S1), even within the cancer (mean = 0.23, SD = 0.15) and control groups (mean = 0.23, SD = 0.20), respectively (Supplementary Table S2). 

**Figure 2:**
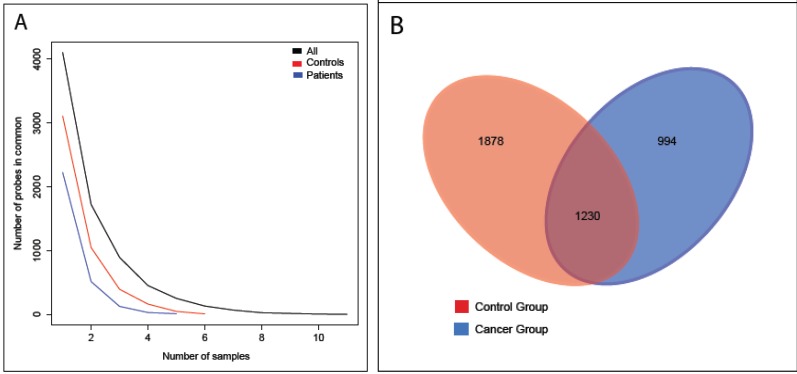
RNA distribution between samples. In (**A**) we show how decreases the number of common genes between an increasing number of samples. Notice that the number of transcripts detected in the control group (red) is larger than the one from cancer group (blue). (**B**) The Venn diagram presents the number of common and unique probes, at least in one sample of the respective group.

Interestingly, there were significantly more detected transcripts in the control samples (mean = 796, SD = 131) than in the cancer samples (mean = 583, SD = 86). The corresponding Mann-Whitney test resulted in a *p*-value of 0.017. The analyzed samples share few common genes, even within groups (see Figure 2A). Specifically, roughly 55% of the transcripts present in at least one cancer sample were detected in at least one control (Figure 2B) and only four genes were spotted in all samples. Additionally, seven genes were present in at least three cancer samples and absent in the control group, and 10 transcripts were identified in at least four samples of control group and absent in the cancer patients (names and annotation of the corresponding genes are detailed in Table 1). As expected, there was some overlap between these transcripts and the list of more differentially expressed transcripts (Supplementary Table S3). Out of these latter, the top 300 were used to build a heatmap that distinguishes between control and cancer samples (Supplementary Figure S2).

**Table 1:** List of the genes that were found in at least four samples of control group and absent from cancer group, and those genes present in at least three samples from cancer group, and absent from control group. The list of genes detected in all the samples of the array is also presented.

**Transcripts present in at least three cancer group samples, and absent in the control group**	
Gene symbol	Gene name
LASS2	Homo sapiens LAG1 homolog, ceramide synthase 2 (LASS2), transcript variant 1, mRNA.
CYB5B	Homo sapiens cytochrome b5 type B (outer mitochondrial membrane) (CYB5B), nuclear gene encoding mitochondrial protein, mRNA.
LOC90624	Homo sapiens hypothetical protein LOC90624 (LOC90624), mRNA.
ST6GALNAC3	Homo sapiens ST6 (alpha-N-acetyl-neuraminyl-2,3-beta-galactosyl-1, 3)-N-acetylgalactosaminide alpha-2,6-sialyltransferase 3 (ST6GALNAC3), mRNA.
HVCN1	Homo sapiens hydrogen voltage-gated channel 1 (HVCN1), transcript variant 1, mRNA.
LOC653107	PREDICTED: Homo sapiens similar to Annexin A8 (Annexin VIII) (Vascular anticoagulant-beta) (VAC-beta), transcript variant 2 (LOC653107), mRNA.
HS.581933	DB298112 BRACE2 Homo sapiens cDNA clone BRACE2040248 3, mRNA sequence
**Present genes in all the samples **	
Gene symbol	Gene name
LOC100130701	PREDICTED: Homo sapiens similar to hCG1657343 (LOC100130701), mRNA.
RHBDL3	Homo sapiens rhomboid, veinlet-like 3 (Drosophila) (RHBDL3), mRNA.
HS.85445	603074330F1 NIH_MGC_119 Homo sapiens cDNA clone IMAGE:5166462 5, mRNA sequence
LOC100129952	PREDICTED: Homo sapiens similar to mCG146274 (LOC100129952), mRNA.
**Transcripts present in at least four control group samples, and absent from the cancer group**	
Gene symbol	Gene name
*ARHGEF39(*C9ORF100)	Homo sapiens chromosome 9 open reading frame 100 (C9orf100), mRNA.
LOC730525	PREDICTED: Homo sapiens hypothetical protein LOC730525 (LOC730525), mRNA.
FRAS1	Homo sapiens Fraser syndrome 1 (FRAS1), transcript variant 3, mRNA.
HBBP1	Homo sapiens hemoglobin, beta pseudogene 1 (HBBP1), non-coding RNA.
NUDT6	Homo sapiens nudix (nucleoside diphosphate linked moiety X)-type motif 6 (NUDT6), transcript variant 1, mRNA.
NEK10	Homo sapiens NIMA (never in mitosis gene a)- related kinase 10 (NEK10), transcript variant 1, mRNA.
KLB	Homo sapiens klotho beta (KLB), mRNA.
LOC51152	PREDICTED: Homo sapiens melanoma antigen (LOC51152), mRNA.
LCN2	Homo sapiens lipocalin 2 (LCN2), mRNA.
LOC650961	PREDICTED: Homo sapiens hypothetical LOC650961 (LOC650961), mRNA.

In order to validate the array results by PCR, we collected a new set of samples and designed primers for a group of transcripts selected according to different criteria, such as whether they were differentially present between groups, within a wide range or intensities in the array, and also some genes that were previously associated with cancer according to CaGE website [26] (Supplementary Table S1). We observed large variability in the transcriptome content among the studied samples, as well as lack of amplification for some mRNAs. In Table 2 we detail the results of PCR and the concordance with the results of the array. 

**Table 2:** Validation of the array. For PCR, each gene was tested in 2 samples for each group, while the number of samples characterized by array was 5 and 6 for cancer and control group, respectively. The four highlighted genes were selected as possible candidates for diagnostic of bladder cancer.

	Positive samples qPCR (%)		Positive samples in array (%)	
GENE	Cancer	Control	Cancer	Control
*AXL*	0	0	0	0
*BCL2*	50	50	0	50
*BCL2L1*	100	50	0	50
*ARHGEF39* *(C9ORF100)*	0	100	0	100
*CYB5B*	100	100	60	0
*DENR*	0	0	20	0
*FOXO3*	0	100	0	33
*GALNT1*	100	0	20	0
*GPR103*	50	50	0	83
*IL18*	50	100	20	33
*LAIR1*	50	100	60	67
*LASS2*	100	0	60	0
*MSH3*	100	100	40	33
*PTGS2*	0	0	0	0
*ROCK2*	100	100	100	83

**Figure 3:**
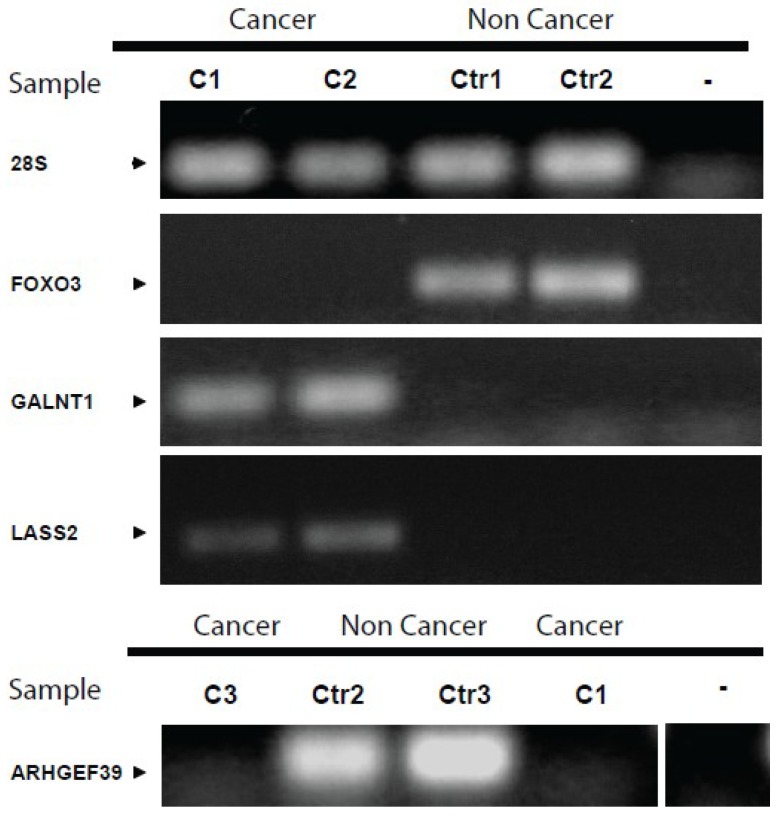
PCR amplification of selected genes in cancer and non-cancer samples. Urinary EVs from cancer patients did not contain *FOXO3* or *ARHGEF39*. However, *GALNT1* and *LASS2* transcripts were present. The opposite result was obtained for samples isolated from non cancer patients. *28S* rRNA transcript was detected in all samples.

Among the tested genes, four genes were present in one group and absent in the other. *LASS2* and *GALNT1* were only present in samples from cancer group, meanwhile *ARHGEF39* (in the array named *Chromosome 9 open reading frame 100* (*C9orf100*)) and *FOXO3* were absent in cancer group and could be detected in control group. The amplification obtained by PCR was visualized in an electrophoresis gel and presented in Figure 3. 

## 4. Discussions and Conclusions

The amount of total RNA we isolated from EVs was very low, ranging from 50 pg to 30 ng, even though we employed a commercial kit that has been reported to maximize the yield of mRNA [27]. We should remark that our samples were frozen, in contrast to the fresh urines employed in the cited work [17]. This could be one of the reasons for the low RNA amount obtained in our study. Moreover, the filtration step that we included is another reason to decrease the total RNA yield, probably because it removes large vesicles from the sample [28]. To avoid vesicles depletion by precipitation of Tamm-Horsfall protein (THP) [29], we did not make an intermediate centrifugation step at 17,000 × *g*. Instead of applying intermediate steps to release EVs from the protein complex trap [30,31], we sediment all types of vesicles present in the urine by ultracentrifugation, after removing cells and cell debris. Therefore, the RNA obtained should represent all cell-free RNA sources in the urine associated with small vesicles. It has been described that the RNA content in cellular debris of urinary patients could be highly degraded, and the transcriptome composition of each sample quite different [32,33]. The RNA size profile that we obtained was enriched in short RNAs, which could correspond to non-coding RNAs or to the product of mRNA fragmentation. We were not able to distinguish ribosomal peaks in the profiles although we successfully detect the presence of *28S* ribosomal gene in all the samples by PCR. The presence of ribosomal fractions and the enrichment in short RNAs both were observed in urinary EVs purified from voided urine [17]. However, the amounts of ribosomal RNA and mRNA obtained in the cited study were larger, and they also reported the presence of DNA.

According to Vesiclepedia [34], the only mRNAs associated with urinary EVs that have been described so far were target-selected genes amplified by PCR [17]. Therefore, this is the first array-based catalogue of the transcriptome carried in urinary EVs. Using mRNA microarray analysis, we identified a number of transcripts within urinary EVs, as well as certain differences between the mRNA content of vesicles obtained from bladder cancer and control samples. 

We have observed great variability among samples, and only 4 transcripts were present in all the urine EVs analyzed by array analysis. In addition, there was a lack of correlation both inter and intra-group (see Supplementary Figure S1 and Supplementary Table S2), reflecting lack of consistency in the mRNA content found in the samples. More interesting, the number of transcripts detected within control samples was larger than in cancer samples (see Figure 2). A possible explanation for this may be a change in the type of vesicles secreted by the epithelia, as it has been described for prostatic cancer [20]. Indeed, exosomes were only present in prostate cancer patients, while prostasomes—vesicles shedding from the outer membrane—were observed only in healthy patients [20]. Therefore, if a similar phenomenon happened in bladder cancer, mRNA content would differ between both types of vesicles. That could explain the results observed in the present study.

The results point to four genes, named *FOXO3*, *GALNT1, ARHGEF39* and *LASS2*, whose presence in urine differs between cancer and non cancer patients. We amplified *ARHGEF39* and *FOXO3* transcripts in non-cancer samples but we could not find them in EVs from cancer patients. *ARHGEF39* is a member of the Dbl-family guanine nucleotide exchange factors (GEFs), which are important activators of Rho GTPases [35]. *FOXO3*, absent in cancer samples, has previously been associated with tumor suppression. Furthermore, it has been described the role of *FOXO3* in cancer and cell proliferation [36,37], and gene expression studies showed that *FOXO3* mRNA expression was lower in cancerous *versus* non cancerous bladder tissues [38]. 

On the contrary, we have found *GALNT1* and *LASS2* only in EVs obtained from cancer patients. Both genes have been reported to be involved in bladder cancer progression [39,40]. *GALNT1* has an important role in transforming growth factor signaling [41], and it has been suggested as a possible biomarker of bladder cancer [42].A similar situation occurs with *LASS2*. Different reports correlate *LASS2* expression with the degree of invasion and recurrence in carcinomas [43,44]. 

The reduced number of samples and the high variability in the transcripts detected for each sample reduce the possible biological meaning obtained from this pilot study. Although the causes for this elevated variability remain unclear and further research is needed to unmask them, our results highlight that certifying biomarkers for disease out of the RNA-content in urinary vesicles will require big cohorts of patients. 

In summary, our work offers a catalogue of transcripts that can be found in urinary EVs. Furthermore, the observed differences between samples from patients with and without bladder cancer suggest that EVs contain valuable information for diagnosis and prognosis. Whether the combination of these four genes may be as useful for diagnosis and prognosis as other genetic signatures obtained from urinary sediment in bladder cancer [11,40,45], would require further investigations.

## Supplementary Files

Supplementary File 1 Supplementary Materials (ZIP, 247 KB)
