# Supplementary material for: A Pilot Study on the Potential of RNA-Associated to Urinary Vesicles as a Suitable Non-Invasive Source for Diagnostic Purposes in Bladder Cancer
Source: Cancers (Basel). 2014 Jan 22;6(1):179–92. doi: 10.3390/cancers6010179 (PMC3980604; doi:10.3390/cancers6010179)

Figure S1. Scatterplot. The upper-right side of the figure displays the correlation between samples. Specifically, 5,000 randomly chosen probes are displayed as points, positioned in the X and Y axes based on their intensities within the corresponding pair of samples. The diagonal is marked in red. Points outside the area delimited by the dashed blue lines have intensities that differ in at least 1 unit of log-intensity between the samples (*i.e.* FC>2). The lower-left side indicates the statistical correlation (Pearson's R) between pairs of samples and how many probes present an FC > 2 at either side.

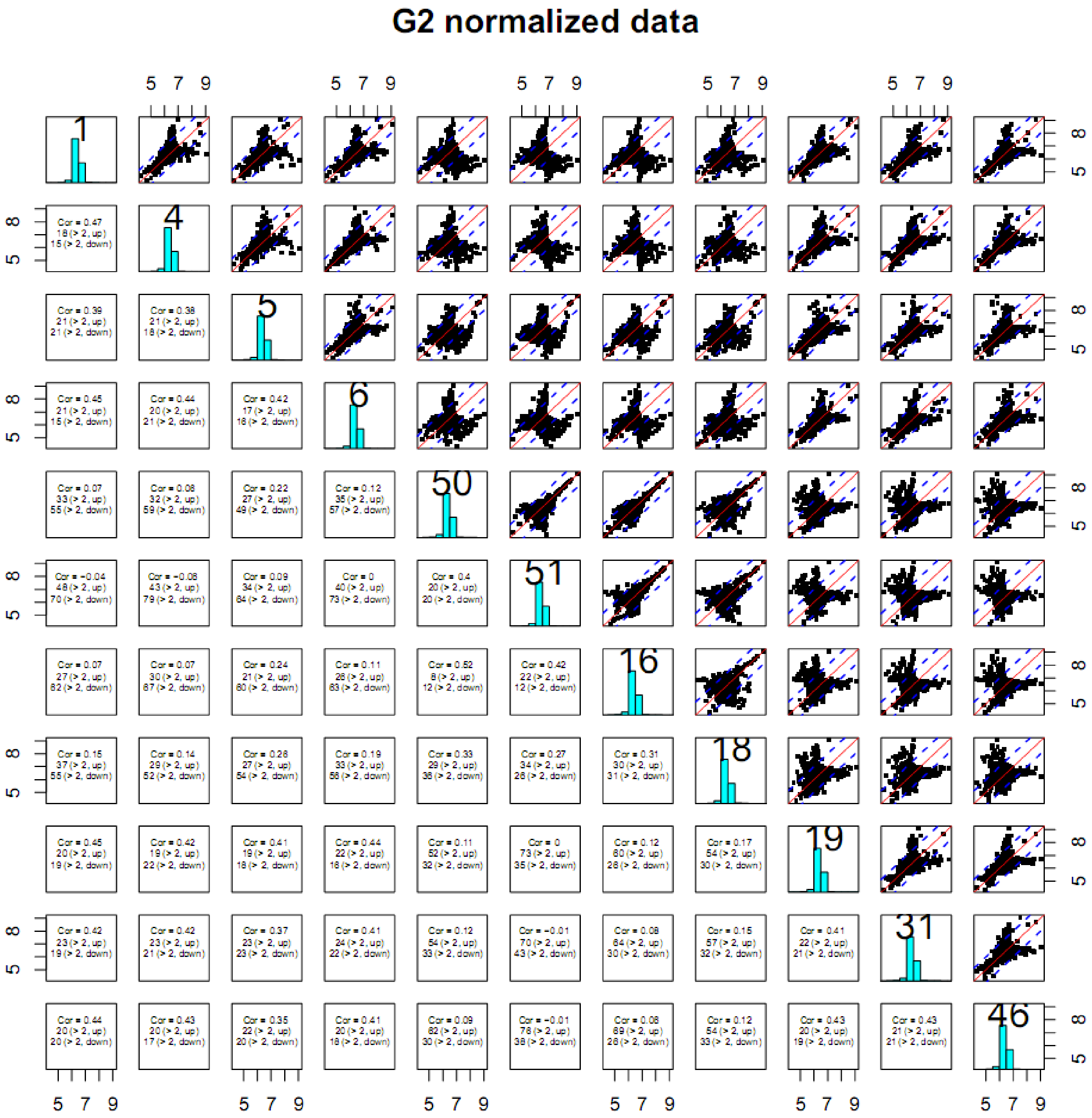

Figure S2. Heatmap with the 300 most significantly differentially expressed transcripts between cancer samples and controls. Samples and transcripts are, respectively, arranged by average linkage clustering. The table with the genes names and values can be found in Supplementary Table S3.

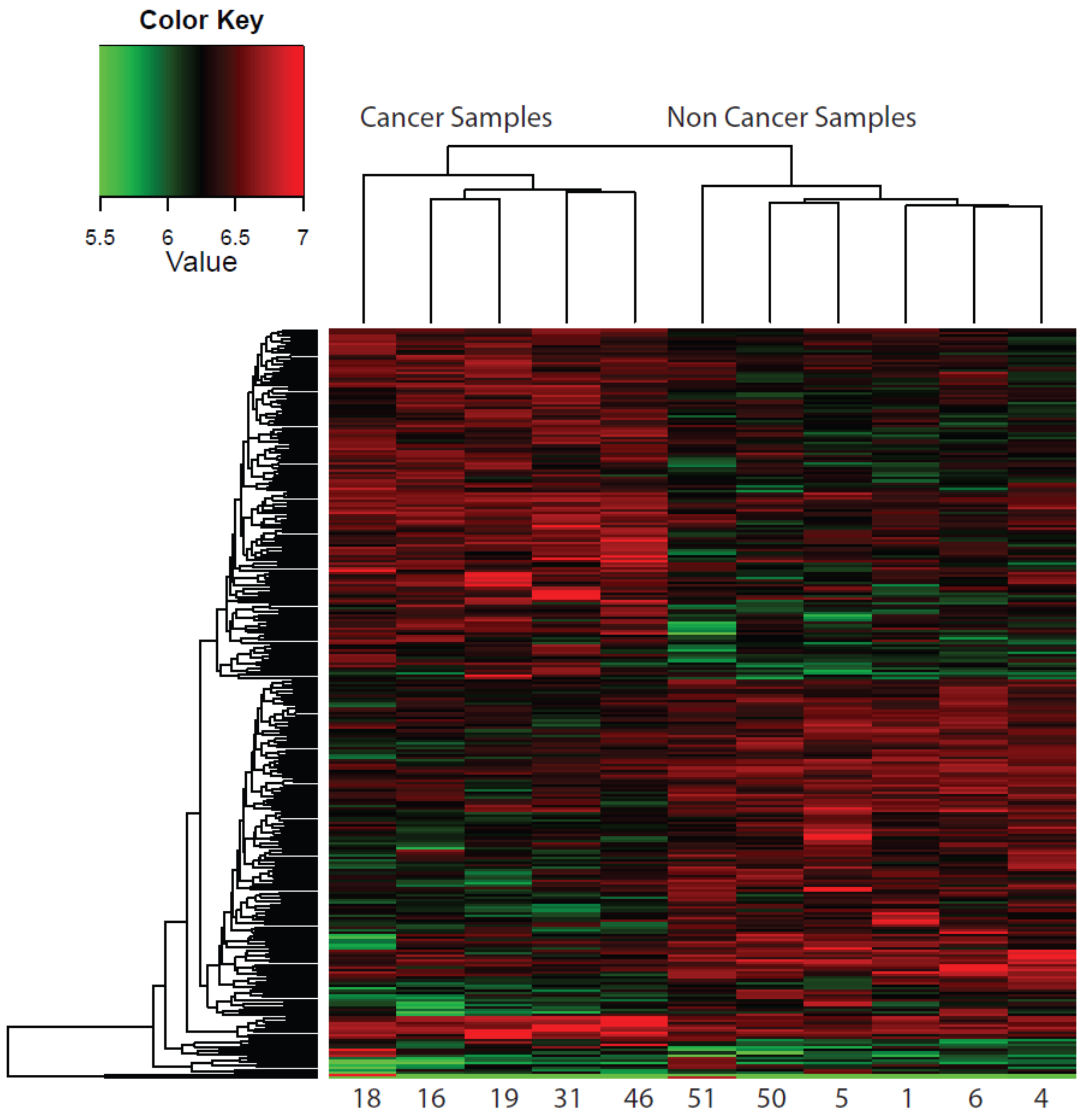

Supplement: Supplementary File 1 — Supplementary Materials (ZIP, 247 KB) [file cancers-06-00179-s001.zip › cancers-43698-supplement-final/cancers-43698-Supplementary Figures S1 and S2.pdf]
